# Visual attention during non-immersive virtual reality balance training in older adults with mild to moderate cognitive impairment: an eye-tracking study

**DOI:** 10.3389/fnagi.2025.1671477

**Published:** 2025-10-21

**Authors:** Marcos Maldonado-Díaz, Gonzalo Jara-Vargas, Felipe González-Seguel

**Affiliations:** 1 Department of Physical Medicine and Rehabilitation, Physical Medicine and Rehabilitation Clínica Alemana Universidad del Desarrollo, Santiago, Chile; 2 Clinica Red Salud Providencia, Santiago, Chile; 3 School of Physical Therapy, Faculty of Medicine, Clínica Alemana, Universidad del Desarrollo, Santiago, Chile

**Keywords:** attention, eye-tracking, cognitive impairment, older adults, virtual reality, balance training

## Abstract

**Background:**

Older adults with cognitive impairment often present with balance deficits, reduced walking speed, and attentional difficulties—particularly in executive function. These challenges increase fall risk and complicate traditional rehabilitation approaches. Eye-tracking technology offers an objective way to evaluate attention by analyzing oculomotor behavior during tasks, but its use in clinical rehabilitation contexts is still limited.

**Objective:**

The aim of this study is to investigate visual attention using eye-tracking metrics during a non-immersive virtual reality-based balance training program in older adults with mild to moderate cognitive impairment.

**Methods:**

This was an exploratory pilot study with a prospective, descriptive cohort, based on a non-controlled, quasi-experimental design of seven older adults with mild to moderate cognitive impairment. Each patient underwent VR-based balance training using Rehametrics^Ⓡ^ software, while their attention was assessed via eye-tracking (Tobii Pro Glasses 2 Refurbished Wireless). Clinical assessments included the Mini-BESTest, Functional Gait Assessment, 6-Minute Walk Test, 4-Meter Walk Test, and Montreal Cognitive Assessment (MoCA). Eye-tracking data focused on fixation patterns, microsaccades, and pupil diameter as indicators of attentional processing.

**Results:**

Patients showed a small numerical increase, without reaching statistical significance in task difficulty progression (*p* = 0.016), lower limb endurance (*p* = 0.016), and single-leg support time (*p* = 0.031). Clinical tests revealed a slight increase, though results were not statistically significant in balance and walking speed (*p* = 0.063). Eye-tracking data indicated increased fixation stability and decreased pupil diameter, suggesting more efficient attention allocation during motor tasks.

**Conclusions:**

Eye-tracking provided valuable metrics into attentional behavior during balance training in older adults with cognitive impairment. Its integration into non-immersive virtual reality rehabilitation may help better understand and address cognitive-motor interactions. Further studies with larger samples are needed to confirm these preliminary findings.

## 1 Introduction

Older adults represent a population particularly vulnerable to motor and cognitive impairment, with approximately 35% experiencing balance or walking impairments by age 70, and only 20% maintaining normal walking capacity by age 85 (Jahn et al., 2010; Middleton et al., 2015). Aging affects not only musculoskeletal control—reducing agonist–antagonist coordination and increasing cortical effort for postural adjustments—but also cognitive functions such as processing speed, attention, memory, and executive control (Casamento-Moran et al., 2017; Van Impe et al., 2013). In individuals with mild cognitive impairment or neurodegenerative conditions such as Parkinson's disease or bilateral vestibulopathy, these deficits are further exacerbated, leading to slower gait, increased fall risk, and impaired dual-task performance (Van Impe et al., 2013; Cavanaugh et al., 2012; Dorsey et al., 2018; Gascuel et al., 2012; Nascimbeni et al., 2010; Ward et al., 2013; Mancino-Moreira et al., 2021; Furman et al., 2010).

These motor deficits are tightly linked to cognitive impairment, especially in executive domains such as inhibition and task switching, which have been shown to predict fall recurrence (Nagy et al., 2020; Taylor et al., 2017). Oculomotor behavior—particularly microsaccades, saccade latency, and fixation duration—has emerged as a sensitive proxy for visual attention and reduced cognitive effort, with abnormalities observed in aging, Parkinson's disease, and Alzheimer's disease (Ebaid and Crewther, 2020; Saftari and Kwon, 2018). Despite this, visual attention and oculomotor control remain underexplored in rehabilitation, even though evidence suggests that attention-based strategies and multisensory stimulation can improve motor and cognitive outcomes in older adults with neurological conditions (Niering and Seifert, 2024; Faria et al., 2020). Moreover, conventional rehabilitation often under-stimulates the cognitive systems involved in motor learning, highlighting the need for more integrative approaches (Ebaid and Crewther, 2020; Saftari and Kwon, 2018).

This study aims to examine whether non-immersive virtual reality (NIVR) balance training can improve balance, visual attention, and motor learning in older adults with cognitive impairments. Using eye-tracking technology, we assessed visual attention through microsaccades and fixation patterns before and after a rehabilitation program. Given that gaze metrics correlate with reduced cognitive effort and executive function (Nyström et al., 2021; Choe et al., 2016; Hayes and Petrov, 2016), we hypothesize that improvements in eye-tracking indicators reflect enhanced attention, leading to better balance control and functional performance (Maldonado-Díaz et al., 2021).

## 2 Methods

### 2.1 Study design

This exploratory pilot study employed a non-controlled, quasi-experimental design with a prospective and descriptive cohort of older adults with mild to moderate cognitive impairment. Patients were enrolled from the outpatient neurorehabilitation unit at the Department of Physical Medicine and Rehabilitation, Clínica Alemana, Santiago, Chile and consisted of balance training using non-immersive virtual reality combined with eye-tracking assessment. Eye-tracking data were collected using the Tobii Pro Glasses 2 Refurbished Wireless, which was available through a six-month research grant awarded via a Latin American competition. Due to this limited access period, seven participants were recruited and completed the full intervention protocol. The study was approved by the “Comité Ético- Científico de Clínica Alemana- Universidad del Desarrollo” (ID: 1167; Protocol Code: 2022-88). This study followed the Transparent Reporting of Evaluations with Nonrandomized Designs (TREND) checklist for transparently reporting non-randomized studies (Des Jarlais et al., 2004). Calibration was performed individually using the Tobii Pro Glasses 2 Refurbished Wireless standard procedure and repeated if necessary; however, “eyes not found” events occasionally occurred, which were attributed not only to calibration but also to participant-related factors such as facial morphology, movement during dynamic tasks, or underlying neurological conditions.

### 2.2 Population

The study included adults over the age of 60 with mild to moderate cognitive impairment (refers to a clinical spectrum of cognitive decline that ranges between normal aging and more severe forms of dementia. It is typically characterized by deficits in one or more cognitive domains—such as memory, attention, executive function, language, or visuospatial skills—that are measurable but do not yet meet the criteria for major neurocognitive disorder) who completed 10 rehabilitation sessions. A non-probabilistic convenience sampling method was used. Patients were screened consecutively and invited to participate voluntarily. All participants were already engaged in conventional rehabilitation programs at the clinic prior to recruitment. The enrollment period lasted 3 months and was aligned with the duration for which the eye-tracking equipment was available for research use. Patients were eligible based on the following criteria: (i) adults over 60 years old with balance disorders, frontotemporal dementia, or Parkinson's disease; (ii) mild to moderate cognitive impairment defined by Montreal Cognitive Assessment (MoCA) score between 10 and 26. Patients were excluded when having (i) visual impairments such as diplopia or spatial hemineglect; (ii) high risk of falls defined by a Mini-BESTest reactive control score of 3 or less; (iii) severe cognitive impairment, defined as a MoCA score below 10; and (iv) refusal to participate in the study.

### 2.3 Intervention protocol

Patients participated in a NIVR balance training program using Rehametrics^®^. Each session included exercises targeting anticipatory postural control, a key component of balance. At the beginning of each task, patients read on-screen instructions and observed a visual demonstration before performing the activity independently. The program automatically adjusted difficulty based on individual performance, modifying elements such as visual distractors, time constraints, and background contrast. Training data—including session count, duration, and single-leg stance characteristics—were recorded. Eye-tracking was used to monitor visual fixation points and gaze duration, while standardized clinical assessments evaluated cognitive function, balance, walking speed, and functional mobility. Supplementary Table S1.

**Figure 1:**
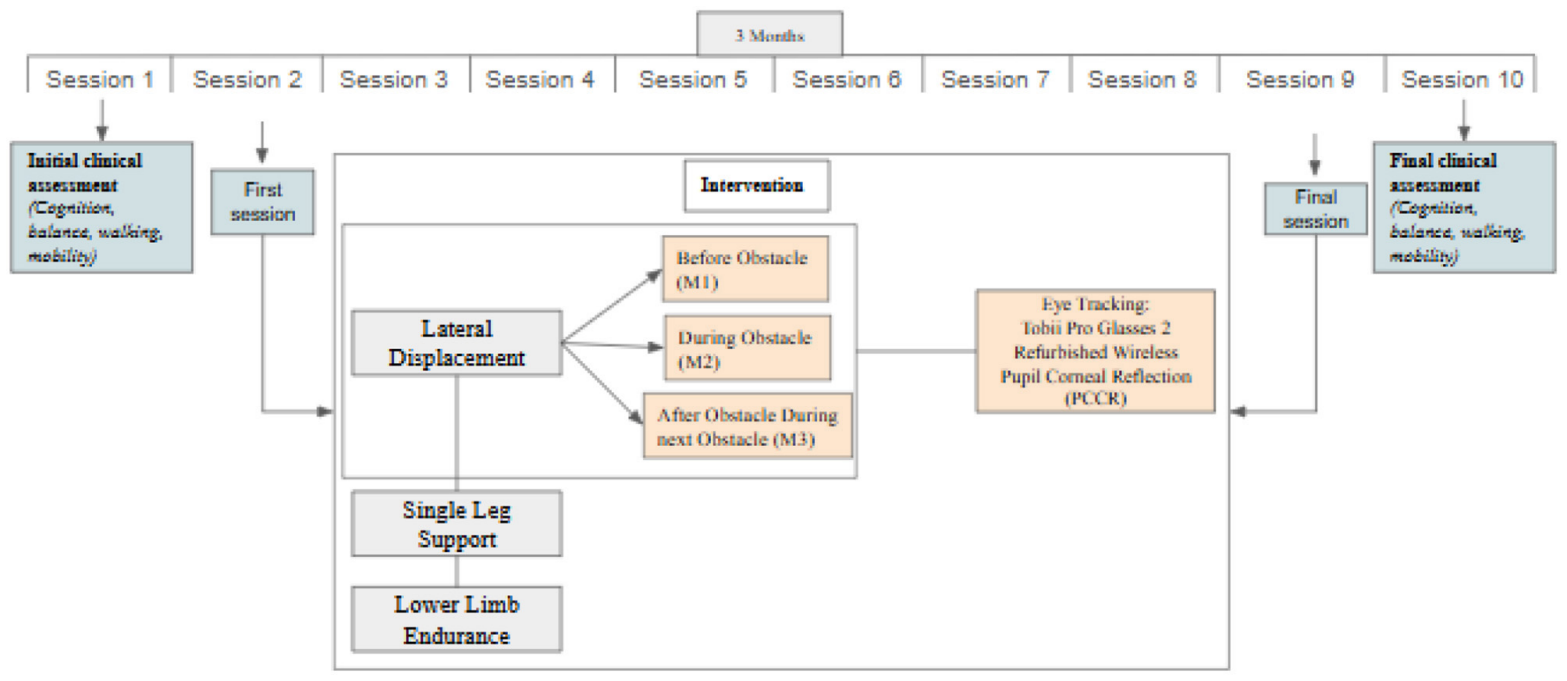
Intervention protocol study timeline.

Each patient completed 10 rehabilitation sessions, each lasting 15 min, combining NIVR balance training with eye tracking (Figure 1). This eye tracker operates by illuminating the eye with a light source to produce distinct reflections, which are captured by a camera. The system identifies reflections from the cornea (glint) and pupil, calculates the vector between these reflections (Pupil-Corneal Reflection or PCCR), and uses its direction along with other geometric features to determine gaze direction. Tobii eye trackers represent an advanced version of traditional remote PCCR-based eye-tracking technology, employing near-infrared illumination to create corneal and pupil reflection patterns captured by image sensors.

The technical setup for the program included a physical space of 3 meters in length by 2 meters in width, a smart TV, and a Wi-Fi connection. The Rehametrics^®^ VR software (version 1.0, Rehametrics SL) was used for the virtual training.

**Figure 2:**
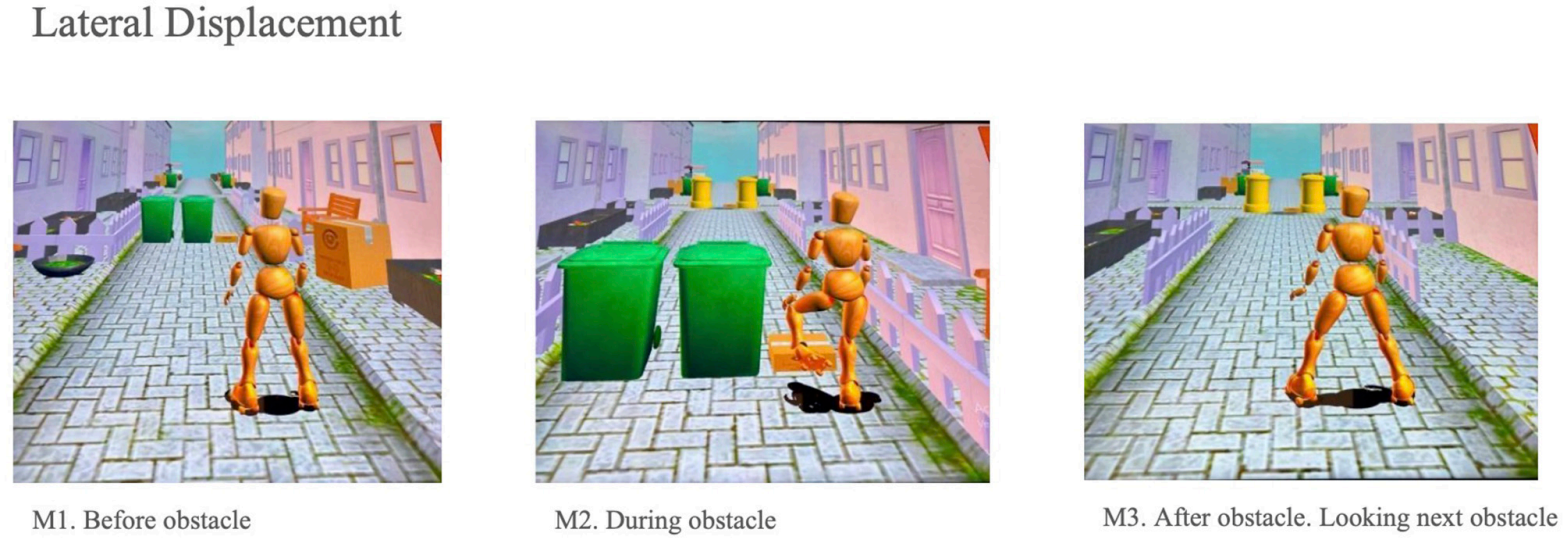
Visual fixation patterns before, during, and after obstacle crossing. Source: Rehametrics (2025).

For eye-tracking data analysis, Tobii Toolbox for MATLAB^®^ was employed, providing an interface between MATLAB^®^ and the Tobii eye trackers via the Tobii Software Development Kit (SDK) in a Windows environment. Since eye-tracking analysis is more accurate with static images, key moments from training sessions on day 2 (T1) and day 9 (T2) were captured. Three stages of a specific exercise were selected for analysis: the patient walking in place while obstacles appear on the floor. The task requires lifting one foot to avoid the obstacle, anticipating the next obstacle, and stepping over it. The aim was to identify how patients visually and motorically respond to this visuomotor challenge and whether their response improved after repeated practice (Figure 2).

For video analysis, two time points were compared: initial (T1) and final (T2). Eye movements were analyzed across stages M1, M2, M3 at T1 and stages M1, M2, M3 at T2. Areas of Interest (AOIs) were drawn on the videos at moments when obstacles appeared, (the patient anticipates an obstacle (M1), passes it and fixes their gaze on the next obstacle (M2) and then anticipates the next obstacle (M3), allowing precise evaluation of gaze patterns relative to the task demands.

### 2.4 Outcomes

The initial assessment included the following clinical measurements: Mini-BESTest (MBT) to evaluate balance and fall risk, Functional Gait Assessment (FGA) to measure walking stability, the 6-Minute Walk Test (6MWT) for walking endurance, and the 4-Meter Walk Test (4-mWT) for walking speed. Based on these assessments, patients were assigned to specific VR exercise clusters. All participants engaged in cluster 5 exercises, which involved walking and balancing tasks with multidirectional changes in base of support.

### 2.5 Analysis

Prospective data were obtained from clinical records, the Virtual Reality rehabilitation report, and the Tobii Pro Glasses 2 Refurbished Wireless eye-tracking device (Lab version 1.241/2024-03-20). Eye movements were analyzed with Tobii Studio software through identification of Areas of Interest (AoI)—defined as specific regions of the image deemed relevant and subject to analysis (Sharafi et al., 2015)—as well as heatmaps and gaze plot graphs. The data recorded by Tobii Studio were exported as flat files and subsequently processed using the Stata 16 statistical software, with no patient-identifying information; each participant was assigned a consecutive study ID at enrollment. Clinical records were maintained in the RedCap database for neurological patients at the SMFR unit. To characterize the patient sample, absolute and relative frequencies were calculated for qualitative variables. For quantitative variables medians (P25-P75) were reported. Spearman's non-parametric correlation test was used to assess potential associations between variables. Visual impairments, visual strategies, and vestibular-visual system adjustments (including microsaccades, gaze angles, pupil data, among others) were analyzed in relation to other variables of interest using Wilcoxon rank-sum test. Independence was tested using chi-square tests or Fisher's exact test, depending on observed frequencies. Finally, Wilcoxon signed-rank tests were used to compare clinical scale scores at baseline and after rehabilitation. All statistical analyses were conducted using Stata 16.

## 3 Results

The intervention was conducted between December 1, 2023, and March 30, 2024, with a total of seven participants enrolled (mean age: 76.9 ± 8.8 years). The sample was predominantly male (86%), and the most frequent diagnosis was Parkinson's disease (57%), followed by balance disorders and frontotemporal dementia (Table 1).

**Table 1:** Demographic characteristics of the study sample.

**Patient**	**Primary diagnosis**	**Sex**	**Age (years)**
Patient 1	Parkinson's disease	Male	72
Patient 2	Balance disorders	Female	80
Patient 3	Frontotemporal dementia	Male	77
Patient 4	Parkinson's disease	Male	75
Patient 5	Balance disorders	Male	92
Patient 6	Parkinson's disease	Male	79
Patient 7	Parkinson's disease	Male	63
Median [P25-P75]			77 [72–80]

Following the non-immersive virtual reality (NIVR) balance training, seven participants (mean age: 76.9 ± 8.8 years; 86% male) demonstrated statistically significant improvements in several motor parameters, including task difficulty progression (*p* = 0.016), lower limb endurance (*p* = 0.016), and single-leg support height (*p* = 0.031). Clinical assessments revealed a small numerical increase, without reaching statistical significance in balance performance as measured by the Mini-BESTest (*p* = 0.063) and in walking speed (p = 0.063), suggesting a slight increase, though results were not statistically significant in functional mobility post-intervention (Table 2).

**Table 2:** Comparison between T1 and T2 in clinical tests, non-immersive virtual reality training outcomes, and eye-tracker findings.

**Session using non-immersive virtual reality**	**T1 - median [P25–P75]**	**T2 median [P25–P75]**	***p*-value**
Difficulty (%)	12 [11–14]	48 [32–50]	0.016
Performance (%)	75 [69–77]	84 [79–88]	0.453
**Lateral displacement**			
Level (max: 45)	1 [1–2]	3 [1–13]	0.375
Hits (%)	44 [33–51]	56 [28–74]	0.453
Errors (%)	56 [26–67]	35 [19–48]	0.453
Average response time (seconds)	11 [11–12]	10 [6–11]	0.125
**Lower limb endurance**			
Level (max: 60)	2 [2–3]	17 [5–22]	0.016
Hits (%)	98 [89–100]	94 [45–98]	0.125
Errors (%)	0 [0–2]	2 [0–6]	0.688
Average response time (seconds)	3 [2–3]	4 [3–7]	0.453
**Single-leg balance**			
Level (max: 21)	2 [2–15]	16 [12–21]	0.031
Hits (%)	88 [77–93]	85 [61–97]	0.999
Errors (%)	0	0	0.999
Average response time (seconds)	5 [4–5]	5 [4–7]	0.999
**Overall result**			
Average step height (cm) (left; right)	12 [0.3–12]	14 [12–28]	0.125
Average step width (cm) (left; right)	10 [7–16]	13 [5–14]	0.453
**Balance (mini-BESTest)**			
Total	20 [14–25]	22 [17–25]	0.063
**Walking stability (functional gait assessment)**			
Total	23 [19–26]	26 [18–28]	0.688
**Cognitive level (MoCA test)**			
Total	26 [21–27]	27 [21–28]	0.250
Walking speed (m/s)	1 [0.6–2]	2 [1–2]	0.063
Walking capacity (6-Minute Walk Test) (meters)	303 [238–364]	308 [290–308]	0.999
Individual standard walking capacity (meters)	420 [400–478]

**Figure 3:**
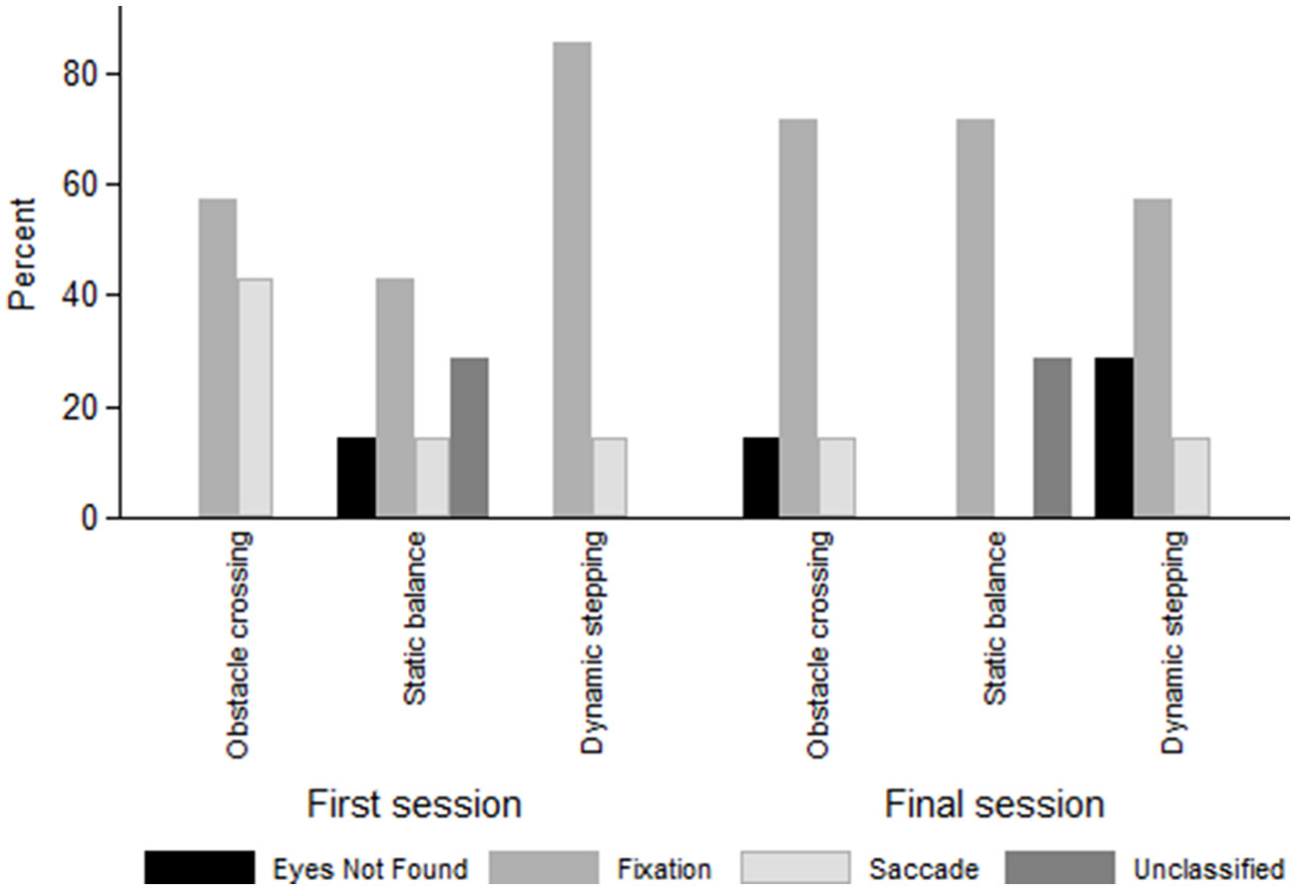
Eye-tracking metrics during obstacle anticipation in virtual reality balance training. This figure shows the distribution of eye-tracking outcomes (fixation stability, microsaccades, and pupil diameter) during obstacle anticipation tasks across two training time points: first session and final session; and three types of exercises performed in non-immersive virtual reality: obstacle crossing, static balance with visual targets, and dynamic stepping with visual anticipation toward the next target. Dynamic stepping specifically requires looking toward the next target, activating anticipatory attention mechanisms. Improvements in attention-related oculomotor behavior are observed between obstacle crossing and static balance.

Eye-tracking analysis showed increased fixation percentages during the second evaluation (T2), exceeding 70% in the initial obstacle anticipation phases. “Eyes Not Found” and “Unclassified” events remained below 30% across all phases and timepoints (Figure 3). Although saccadic activity varied between sessions, no statistically significant differences were observed in ocular metrics between pre- and post-intervention measurements. These findings are summarized in Supplementary Table S2 and illustrated in Figure 2.

## 4 Discussion

This exploratory pilot study revealed a small numerical increase, without reaching statistical significance in balance, walking speed, and visual attention among older adults with neurodegenerative conditions by receiving non-immersive virtual reality balance training. These outcomes were supported by both clinical assessments and eye-tracking data, which showed reductions in median values and variability across several parameters. A trend toward improvement was observed in pupil diameter, which decreased at T2 compared to T1 (Supplementary Table S2). However, the initial measurement, exceeding 9 mm, seems abnormally high for this population and likely reflects a calibration issue, as such values are rarely reported in the literature for aging adults under standard lighting conditions (Telek et al., 2018; Karatekin, 2007). Even so, the overall trend of decreasing pupil size may suggest reduced cognitive effort and greater task familiarity, which has been described in previous studies on cognitive aging (El Haj, 2024).

We also observed a shift from frequent saccades at T1 to longer fixations at T2, suggesting a possible adjustment in visual strategies. This pattern may indicate a more efficient allocation of attention, consistent with observations in visuomotor learning research, where repeated exposure to tasks tends to stabilize fixations and reduce superfluous eye movements (Marino and Mazer, 2016). From a physiological standpoint, these adaptations could reflect preliminary evidence of changes in attentional filtering and emotional regulation, functions previously linked with smaller pupil diameters during cognitive tasks (Manohar et al., 2017; Lavin et al., 2014; Varazzani et al., 2015; Kahneman and Beatty, 1966; Gilzenrat et al., 2010; van der Wel and van Steenbergen, 2018). Additionally, participants' ability to sustain visual focus during dynamic, full-body movement points to the cognitive demands embedded in NIVR balance training (Gkintoni et al., 2025).

Taken together, these oculomotor changes provide additional context to the clinical outcomes, highlighting the potential value of integrating eye-tracking into rehabilitation protocols. While some rapid fluctuations in pupil size were noted, these are unlikely to be explained solely by changes in lighting or task structure and may instead relate to attentional processes (Grujic et al., 2024). At the same time, the slight increase in “Eyes Not Found” events at T2 suggests the presence of fatigue or calibration issues, underscoring the need for more robust standardization in future protocols.

Beyond the specific findings of this pilot study, non-immersive VR has been explored in prior research as a rehabilitation tool, with reported benefits for balance, gait, and cognition (Rehametrics, 2025; Dockx et al., 2016; Laver et al., 2017; Wulf, 2013; Hunt et al., 2017; Sugianto et al., 2025; Huang et al., 2024). Our results cannot confirm such efficacy, but they are consistent with these broader trends and may support the rationale for further trials. Importantly, the motivational aspects of VR training, observed in our participants' engagement across increasing task difficulty, suggest that this approach could complement conventional rehabilitation strategies. However, given the exploratory design and limited sample, the present findings should be considered preliminary and interpreted with caution. Future randomized controlled trials with larger samples are needed to determine whether the trends observed here translate into clinically meaningful benefits (Dockx et al., 2016; Laver et al., 2017; Tortora et al., 2024).

## 5 Limitations

This study has several limitations. The small sample size restricts generalizability and statistical power. Additionally, the heterogeneity of neurological diagnoses introduces variability that may obscure condition-specific effects. In part, this was due to the exploratory nature of the study and to recruitment and equipment availability constraints. Therefore, the findings should be considered preliminary and orientative, and future studies with more homogeneous cohorts are needed to analyze diagnosis-specific effects. Moreover, the absence of additional physiological measures, such as EEG or galvanic skin response (GSR), limits interpretation of arousal and emotional engagement (Gkintoni et al., 2025; Sugianto et al., 2025).

It should be noted that “eyes not found” events may have affected data capture; these were likely related not only to calibration issues but also to participant-specific factors such as facial features, movement during dynamic tasks, or neurological conditions, which may have influenced eye-tracking accuracy.

Baseline pupil diameter values should be interpreted with caution, as calibration issues with the Tobii Pro Glasses 2 Refurbished Wireless and participant-specific factors may have influenced accuracy; future studies will address this with stricter pre-recruitment procedures, more controlled environments, and improved data collection protocols.

The absence of formal correction for multiple comparisons increases the potential risk of Type I error, which should be considered when interpreting the findings.

Because effect sizes and confidence intervals were not calculated, borderline p-values should be interpreted with caution and considered non-significant within the exploratory context of this pilot study.

Calibration of eye-tracking devices is generally more reliable under static, seated conditions with minimal head movement; because our participants performed dynamic balance tasks, pupil diameter results should be interpreted with caution and considered exploratory only.

In summary, the main limitations can be outlined as follows: (i) very small sample size; (ii) diagnostic heterogeneity; (iii) absence of a control group; (iv) possible calibration/technical issues; and (v) limited generalizability. These factors highlight the exploratory character of the study and reinforce the need for larger, more stratified cohorts in future research.

### 5.1 Clinical and translational implications

Clinically, the results of this study suggest that integrating visual-attentional strategies and oculomotor assessments could enhance motor learning. For example, external focus of attention (EFA) strategies—shown to improve performance in both musculoskeletal and neurological populations—could be applied alongside eye-tracking feedback to promote optimal motor planning (Wulf, 2013; Hunt et al., 2017). In keeping with emerging research in neurodegenerative conditions, our exploratory findings underscore the potential of eye-tracking not just as a research tool but as a clinical adjunct. In Parkinson's disease, eye-tracking has been validated as a reliable, cost-effective method for detection, cognitive assessment, and rehabilitation (Diotaiuti et al., 2025). Further, a recent systematic review highlights that metrics such as saccade velocity, fixation duration, and pupillary changes correlate with disease severity—with increasing scalability through machine learning and VR integration (Culicetto et al., 2025). Importantly, Dalbro et al. (2025) demonstrated the repeatability and reliability of eye-movement metrics (fixations, saccades) in Parkinson's patients, laying the groundwork for their use in clinical monitoring. From a practical standpoint, ensuring that impaired patients visually attend to therapeutic tasks is crucial for efficient therapy—eye-tracking provides therapists with objective, real-time confirmation of attention, helping to avoid wasted effort. As accessible eye-tracking systems become more widespread, they offer opportunities for personalized, attention-informed rehabilitation protocols in outpatient or community settings. Future studies should explore these integrative approaches over longer periods to assess their impact on functional recovery and quality of life.

## 6 Conclusions

A small group of participants with mild to moderate cognitive impairments showed a small numerical increase, without reaching statistical significance in balance, walking speed, and visual attention by receiving NIVR balance training combined with eye-tracking. Clinical assessments showed a non-significant change compared to baseline in visuospatial function and postural control, marked by a shift from rapid saccades to longer gaze fixations—suggesting improved visual strategies. These findings support the integration of attentional and oculomotor components in rehabilitation and highlight the potential of this approach as an accessible, personalized tool for motor-cognitive recovery in individuals with neurodegenerative conditions. As an exploratory pilot study, these results should be interpreted with caution but provide a foundation for larger and controlled follow-up trials.

## Data Availability

The datasets presented in this study can be found in online repositories. The names of the repository/repositories and accession number(s) can be found in the article/Supplementary material.

## References

[B1] Casamento-Moran A. Chen Y. T. Lodha N. Yacoubi B. Christou E. A. (2017). Motor plan differs for young and older adults during similar movements. J. Neurophysiol. 117, 1483–1488. doi: 10.1152/jn.00640.2016 28077666 PMC5376608

[B2] Cavanaugh J. T. Ellis T. D. Earhart G. M. Ford M. P. Foreman K. B. Dibble L. E. . (2012). Capturing ambulatory activity decline in Parkinson's disease. J. Neurol. Phys. Ther. 36, 51–57. doi: 10.1097/NPT.0b013e318254ba7a 22592060 PMC3934648

[B3] Choe K. W. Blake R. Lee S. H. (2016). Pupil size dynamics during fixation impact the accuracy and precision of video-based gaze estimation. Vis. Res. 118, 48–59. doi: 10.1016/j.visres.2014.12.018 25578924

[B4] Culicetto L. Cardile D. Marafioti G. Lo Buono V. Ferraioli F. Massimino S. . (2025). Recent advances (2022-2024) in eye-tracking for Parkinson's disease: a promising tool for diagnosing and monitoring symptoms. Front. Aging Neurosci. 17:1534073. doi: 10.3389/fnagi.2025.1534073 40469846 PMC12133813

[B5] Dalbro S. E. J. Elsais A. Rydning S. L. Toft M. Kerty E. Larsen S. E. . (2025). Repeatability, reliability, and stability of eye movement measurements in Parkinson's disease, cerebellar ataxia, and healthy adults. Front. Neurol. (2025) 16:1556314. doi: 10.3389/fneur.2025.1556314 40356635 PMC12068062

[B6] Des Jarlais D. C. Lyles C. Crepaz N. TREND Group (2004). Improving the reporting quality of nonrandomized evaluations of behavioral and public health interventions: the TREND statement. Am. J. Public Health 94, 361–366. doi: 10.2105/AJPH.94.3.361 14998794 PMC1448256

[B7] Diotaiuti P. Marotta G. Di Siena F. Vitiello S. Di Prinzio F. Rodio A. . (2025). Eye tracking in Parkinson's disease: a review of oculomotor markers and clinical applications. Brain Sci. 15:362. doi: 10.3390/brainsci15040362 40309816 PMC12025636

[B8] Dockx K. Bekkers E. M. Van den Bergh V. Ginis P. Rochester L. Hausdorff J. M. . (2016). Virtual reality for rehabilitation in Parkinson's disease. Cochrane Database Syst Rev. 12, CD010760. doi: 10.1002/14651858.CD010760.pub2 PMC6463967 28000926

[B9] Dorsey E. R. Elbaz A. Nichols E. Abbasi N. Abd-Allah F. Abdelalim A. . (2018). Global, regional, and national burden of Parkinson's disease, 1990–2016: a systematic analysis for the Global Burden of Disease Study (2016). Lancet Neurol. 17, 939–953. doi: 10.1016/S1474-4422(18)30295-3 30287051 PMC6191528

[B10] Ebaid D. Crewther S. G. (2020). The contribution of oculomotor functions to rates of visual information processing in younger and older adults. Sci. Rep. 10:10129. doi: 10.1038/s41598-020-66773-5 32576849 PMC7311387

[B11] El Haj M. (2024). Pupillometry as tool to assess cognitive and affective processing in aging. Brain Disord. 14:100129. doi: 10.1016/j.dscb.2024.100129

[B12] Faria A. L. Pinho M. S. Bermúdez I. Badia S. (2020). A comparison of two personalization and adaptive cognitive rehabilitation approaches: a randomized controlled trial with chronic stroke patients. J. Neuroeng. Rehabil. 17:78. doi: 10.1186/s12984-020-00691-5 32546251 PMC7298954

[B13] Furman J. M. Raz Y. Whitney S. L. (2010). Geriatric vestibulopathy assessment and management. Curr. Opin. Otolaryngol. Head Neck Surg. 18, 386–391. doi: 10.1097/MOO.0b013e32833ce5a6 20613528 PMC4879828

[B14] Gascuel J. D. Payno H. Schmerber S. Martin O. (2012). Immersive virtual environment for visuo-vestibular therapy: preliminary results. Stud. Health Technol. Inform. 181, 187–191. doi: 10.3233/978-1-61499-121-2-187 22954853

[B15] Gilzenrat M. S. Nieuwenhuis S. Jepma M. Cohen J. D. (2010). Pupil diameter tracks changes in control state predicted by the adaptive gain theory of locus coeruleus function. Cogn. Affect. Behav. Neurosci. 10, 252–269. doi: 10.3758/CABN.10.2.252 20498349 PMC3403821

[B16] Gkintoni E. Aroutzidis A. Antonopoulou H. Halkiopoulos C. (2025). From neural networks to emotional networks: a systematic review of EEG-based emotion recognition in cognitive neuroscience and real-world applications. Brain Sci. 15:220. doi: 10.3390/brainsci15030220 40149742 PMC11940461

[B17] Grujic N. Polania R. Burdakov D. (2024). Neurobehavioral meaning of pupil size. Neuron 112, 3381–3395. doi: 10.1016/j.neuron.2024.05.029 38925124

[B18] Hayes T. R. Petrov A. A. (2016). Mapping and correcting the influence of gaze position on pupil size measurements. Behav. Res. Methods 48, 510–527. doi: 10.3758/s13428-015-0588-x 25953668 PMC4637269

[B19] Huang W. Y. Chang S. T. Lee C. H. Liou I. H. Cherng R. J. (2024). Effects of virtual reality on the balance performance of older adults: a systematic review and meta-analysis. J. Phys. Ther. Sci. 36, 457–470. doi: 10.1589/jpts.36.457 39092409 PMC11290862

[B20] Hunt C. Paez A. Folmar E. (2017). The impact of attentional focus on the treatment of musculoskeletal and movement disorders. Int. J. Sports Phys. Ther. 12, 901–907. doi: 10.26603/ijspt20170901 29158952 PMC5675366

[B21] Jahn K. Zwergal A. Schniepp R. (2010). Gait disturbances in old age: classification, diagnosis, and treatment from a neurological perspective. Dtsch. Arzteblatt. Int. 107, 306–315. doi: 10.3238/arztebl.2010.0306 20490346 PMC2872829

[B22] Kahneman D. Beatty J. (1966). Pupil diameter and load on memory. Science 154, 1583–1585. doi: 10.1126/science.154.3756.1583 5924930

[B23] Karatekin C. (2007). Eye tracking studies of normative and atypical development. Dev. Rev. 27, 283–348. doi: 10.1016/j.dr.2007.06.006

[B24] Laver K. E. Lange B. George S. Deutsch J. E. Saposnik G. Crotty M. . (2017). Virtual reality for stroke rehabilitation. Cochrane Database Syst. Rev. 11:CD008349. doi: 10.1002/14651858.CD008349.pub4 29156493 PMC6485957

[B25] Lavin C. San Martín R. Rosales Jubal E. (2014). Pupil dilation signals uncertainty and surprise in a learning gambling task. Front. Behav. Neurosci. 7:218. doi: 10.3389/fnbeh.2013.00218 24427126 PMC3879532

[B26] Maldonado-Díaz M. Vargas P. Vasquez R. Gonzalez-Seguel F. Rivero B. Hidalgo-Cabalín V. . (2021). Teleneurorehabilitation program (virtual reality) for patients with balance disorders: descriptive study. BMC Sports Sci. Med. Rehabil. 13:83. doi: 10.1186/s13102-021-00314-z 34340687 PMC8330090

[B27] Mancino-Moreira F. Rueda A. Esteban-Sanchez J. Martin-Sanz E. (2021). Clinical subtypes and vHIT parameters in a population with bilateral vestibulopathy. Front. Neurol. 12:673974. doi: 10.3389/fneur.2021.673974 34163428 PMC8216236

[B28] Manohar S. G. Finzi R. D. Drew D. Husain M. (2017). distinct motivational effects of contingent and noncontingent rewards. Psychol. Sci. 28, 1016–1026. doi: 10.1177/0956797617693326 28488927 PMC5510684

[B29] Marino A. C. Mazer J. A. (2016). Perisaccadic updating of visual representations and attentional states: linking behavior and neurophysiology. Front. Syst. Neurosci. 10:3. doi: 10.3389/fnsys.2016.00003 26903820 PMC4743436

[B30] Middleton A. Fritz S. L. Lusardi M. (2015). Walking speed: the functional vital sign. J. Aging Phys. Act. 23, 314–322. doi: 10.1123/japa.2013-0236 24812254 PMC4254896

[B31] Nagy B. Czigler I. File D. Gaál Z. A. (2020). Can irrelevant but salient visual cues compensate for the age-related decline in cognitive conflict resolution?—an ERP study. PLoS ONE 15:e0233496. doi: 10.1371/journal.pone.0233496 32433679 PMC7239486

[B32] Nascimbeni A. Gaffuri A. Penno A. Tavoni M. (2010). Dual task interference during gait in patients with unilateral vestibular disorders. J. Neuroeng. Rehabil. 7:47. doi: 10.1186/1743-0003-7-47 20854671 PMC2949709

[B33] Niering M. Seifert J. (2024). The effects of visual skills training on cognitive and executive functions in stroke patients: a systematic review with meta-analysis. J. Neuroeng. Rehabil. 21:41. doi: 10.1186/s12984-024-01338-5 38532485 PMC10967170

[B34] Nyström M. Niehorster D. C. Andersson R. Hooge I. (2021). The Tobii Pro Spectrum: a useful tool for studying microsaccades? Behav. Res. Methods 53, 335–353. doi: 10.3758/s13428-020-01430-3 32705656 PMC7880983

[B35] Rehametrics (2025). Software for Physiotherapy. Valencia (SPAIN). Available online at: https://rehametrics.com/software-fisioterapia (Accessed 2025).

[B36] Saftari L. N. Kwon O. S. (2018). Ageing vision and falls: a review. J. Physiol. Anthropol. 37:11. doi: 10.1186/s40101-018-0170-1 29685171 PMC5913798

[B37] Sharafi Z. Shaffer T. Sharif B. Guéhéneuc Y. G. (2015). Eye-Tracking Metrics in Software Engineering.

[B38] Sugianto M. Zhou Y. Qiu J. (2025). Eye tracking-based dual task in rehabilitation of motor and cognitive function in post-stroke patients: a literature review. Bull. Fac. Phys. Ther. 30:33. doi: 10.1186/s43161-025-00295-x

[B39] Taylor M. E. Lord S. R. Delbaere K. Kurrle S. E. Mikolaizak A. S. Close J. C. T. . (2017). Reaction time and postural sway modify the effect of executive function on risk of falls in older people with mild to moderate cognitive impairment. Am. J. Geriatr. Psychiatry Off. J. Am. Assoc. Geriatr. Psychiatry 25, 397–406. doi: 10.1016/j.jagp.2016.10.010 28063853

[B40] Telek H. H. Erdol H. Turk A. (2018). The effects of age on pupil diameter at different light amplitudes. Beyoglu Eye J. 3, 80–85. doi: 10.14744/bej.2018.43534

[B41] Tortora C. Di Crosta A. La Malva P. Prete G. Ceccato I. Mammarella N. . (2024). Virtual reality and cognitive rehabilitation for older adults with mild cognitive impairment: a systematic review. Ageing Res Rev. 93:102146. doi: 10.1016/j.arr.2023.102146 38036103

[B42] van der Wel P. van Steenbergen H. (2018). Pupil dilation as an index of effort in cognitive control tasks: a review. Psychon. Bull. Rev. 25, 2005–2015. doi: 10.3758/s13423-018-1432-y 29435963 PMC6267528

[B43] Van Impe A. Bruijn S. M. Coxon J. P. Wenderoth N. Sunaert S. Duysens J. . (2013). Age-related neural correlates of cognitive task performance under increased postural load. Age 35, 2111–2124. doi: 10.1007/s11357-012-9499-2 23274853 PMC3824995

[B44] Varazzani C. San-Galli A. Gilardeau S. Bouret S. (2015). Noradrenaline and dopamine neurons in the reward/effort trade-off: a direct electrophysiological comparison in behaving monkeys. J. Neurosci. Off. J. Soc. Neurosci. 35, 7866–7877. doi: 10.1523/JNEUROSCI.0454-15.2015 25995472 PMC6795183

[B45] Ward B. K. Agrawal Y. Hoffman H. J. Carey J. P. Della Santina C. C. (2013). Prevalence and impact of bilateral vestibular hypofunction: results from the 2008 US National Health Interview Survey. JAMA Otolaryngol.– Head Neck Surg. 139, 803–810. doi: 10.1001/jamaoto.2013.3913 23949355 PMC4839981

[B46] Wulf G. (2013). Attentional focus and motor learning: a review of 15 years. Int. Rev. Sport Exerc. Psychol. 6, 77–104. doi: 10.1080/1750984X.2012.723728

