# Correction: Visual attention during non-immersive virtual reality balance training in older adults with mild to moderate cognitive impairment: an eye-tracking study

**DOI:** 10.3389/fnagi.2025.1737079

**Published:** 2025-12-02

**Authors:** Marcos Maldonado-Díaz, Gonzalo Jara-Vargas, Felipe González-Seguel

**Affiliations:** 1 Department of Physical Medicine and Rehabilitation, Physical Medicine and Rehabilitation Clínica Alemana Universidad del Desarrollo, Santiago, Chile; 2 Clinica Red Salud Providencia, Santiago, Chile; 3 School of Physical Therapy, Faculty of Medicine, Clínica Alemana, Universidad del Desarrollo, Santiago, Chile

**Keywords:** attention, eye-tracking, cognitive impairment, older adults, virtual reality, balance training

In the published article, the phrase “Department of Physical Medicine and Rehabilitation” was erroneously included in affiliation 3 as written in the author list. The correct affiliation 3 should be: “3. School of Physical Therapy, Faculty of Medicine, Clínica Alemana, Universidad del Desarrollo, Santiago, Chile”.

In the published article, there was a mistake in Figure 1 as published where “Tobii Pro Tx300” was erroneously included instead of “Tobii Pro Glasses 2”. The corrected Figure 1 appears below.

**Figure 1:**
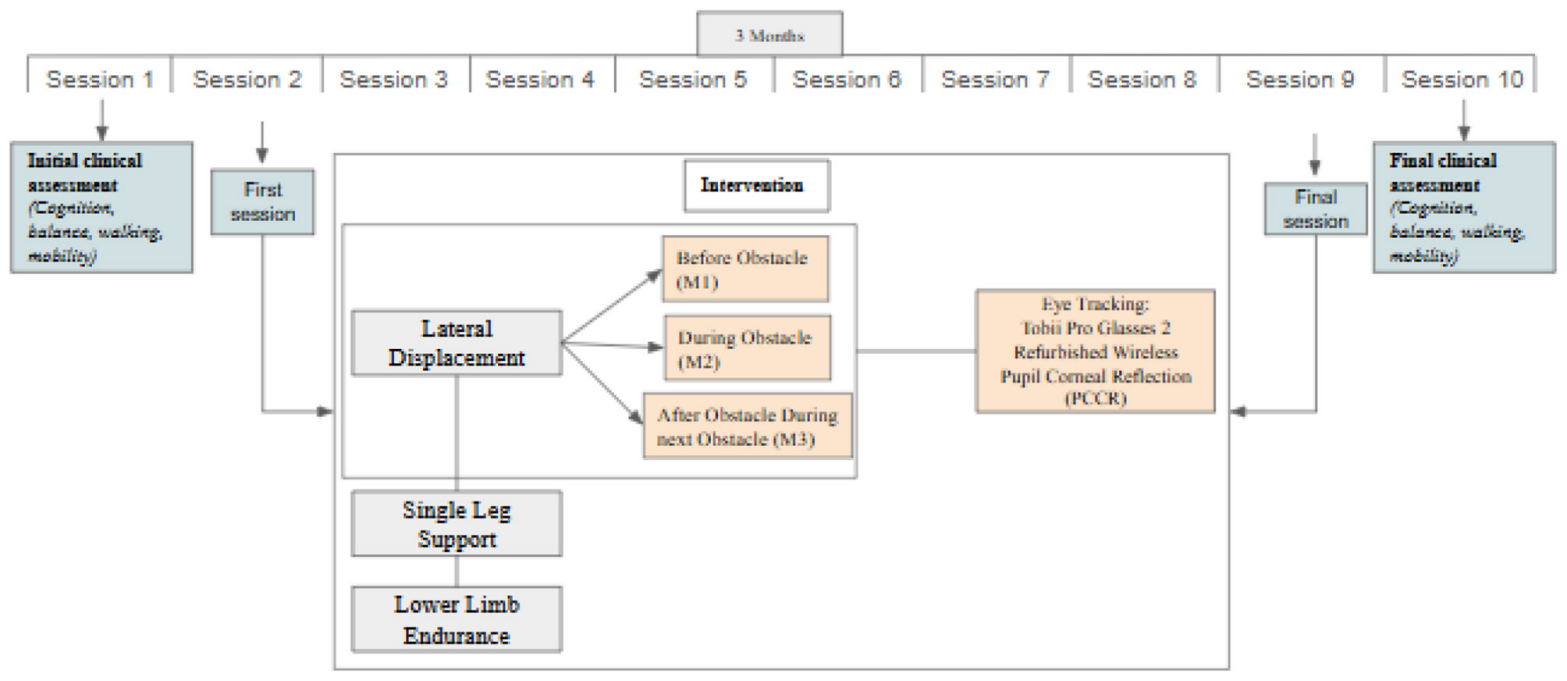
Intervention protocol study timeline.

In the published article, in the abstract, “Tobii Pro TX300” was erroneously included instead of “Tobii Pro Glasses 2 Refurbished Wireless” The updated abstract appears below.

“**Background:** Older adults with cognitive impairment often present with balance deficits, reduced walking speed, and attentional difficulties—particularly in executive function. These challenges increase fall risk and complicate traditional rehabilitation approaches. Eye-tracking technology offers an objective way to evaluate attention by analyzing oculomotor behavior during tasks, but its use in clinical rehabilitation contexts is still limited.

**Objective:** The aim of this study is to investigate visual attention using eye-tracking metrics during a non-immersive virtual reality-based balance training program in older adults with mild to moderate cognitive impairment.

**Methods:** This was an exploratory pilot study with a prospective, descriptive cohort, based on a non-controlled, quasi-experimental design of seven older adults with mild to moderate cognitive impairment. Each patient underwent VR-based balance training using Rehametrics^Ⓡ^ software, while their attention was assessed via eye-tracking (Tobii Pro Glasses 2 Refurbished Wireless). Clinical assessments included the Mini-BESTest, Functional Gait Assessment, 6-Minute Walk Test, 4-Meter Walk Test, and Montreal Cognitive Assessment (MoCA). Eye-tracking data focused on fixation patterns, microsaccades, and pupil diameter as indicators of attentional processing.

**Results:** Patients showed a small numerical increase, without reaching statistical significance in task difficulty progression (*p* = 0.016), lower limb endurance (*p* = 0.016), and single-leg support time (*p* = 0.031). Clinical tests revealed a slight increase, though results were not statistically significant in balance and walking speed (*p* = 0.063). Eye-tracking data indicated increased fixation stability and decreased pupil diameter, suggesting more efficient attention allocation during motor tasks.

**Conclusions:** Eye-tracking provided valuable metrics into attentional behavior during balance training in older adults with cognitive impairment. Its integration into non-immersive virtual reality rehabilitation may help better understand and address cognitive-motor interactions. Further studies with larger samples are needed to confirm these preliminary findings.”

In the published article, in the body text, “Tobii Pro TX300” was erroneously included instead of “Tobii Pro Glasses 2 Refurbished Wireless”. A correction has been made to section **2 Methods**, *2.1 Study design*, paragraph 1 as below:

“This exploratory pilot study employed a non-controlled, quasi-experimental design with a prospective and descriptive cohort of older adults with mild to moderate cognitive impairment. Patients were enrolled from the outpatient neurorehabilitation unit at the Department of Physical Medicine and Rehabilitation, Clínica Alemana, Santiago, Chile and consisted of balance training using non-immersive virtual reality combined with eye-tracking assessment. Eye-tracking data were collected using the Tobii Pro Glasses 2 Refurbished Wireless, which was available through a six-month research grant awarded via a Latin American competition. Due to this limited access period, seven participants were recruited and completed the full intervention protocol. The study was approved by the “Comité Ético- Científico de Clínica Alemana- Universidad del Desarrollo” (ID: 1167; Protocol Code: 2022-88). This study followed the Transparent Reporting of Evaluations with Nonrandomized Designs (TREND) checklist for transparently reporting non-randomized studies (Des Jarlais et al., 2004). Calibration was performed individually using the Tobii Pro Glasses 2 Refurbished Wireless standard procedure and repeated if necessary; however, “eyes not found” events occasionally occurred, which were attributed not only to calibration but also to participant-related factors such as facial morphology, movement during dynamic tasks, or underlying neurological conditions.”

In the published article, in the body text, “Tobii Pro TX300” was erroneously included instead of “Tobii Pro Glasses 2 Refurbished Wireless”. A correction has been made to section **2 Methods**, *2.5 Analysis*, paragraph 1 as below:

“Prospective data were obtained from clinical records, the Virtual Reality rehabilitation report, and the Tobii Pro Glasses 2 Refurbished Wireless eye-tracking device (Lab version 1.241/2024-03-20). Eye movements were analyzed with Tobii Studio software through identification of Areas of Interest (AoI)—defined as specific regions of the image deemed relevant and subject to analysis (Sharafi et al., 2015)—as well as heatmaps and gaze plot graphs. The data recorded by Tobii Studio were exported as flat files and subsequently processed using the Stata 16 statistical software, with no patient-identifying information; each participant was assigned a consecutive study ID at enrollment. Clinical records were maintained in the RedCap database for neurological patients at the SMFR unit. To characterize the patient sample, absolute and relative frequencies were calculated for qualitative variables. For quantitative variables medians (P25-P75) were reported. Spearman's non-parametric correlation test was used to assess potential associations between variables. Visual impairments, visual strategies, and vestibular-visual system adjustments (including microsaccades, gaze angles, pupil data, among others) were analyzed in relation to other variables of interest using Wilcoxon rank-sum test. Independence was tested using chi-square tests or Fisher's exact test, depending on observed frequencies. Finally, Wilcoxon signed-rank tests were used to compare clinical scale scores at baseline and after rehabilitation. All statistical analyses were conducted using Stata 16.”

In the published article, in the body text, “Tobii Glasses 2” was erroneously included instead of “Tobii Pro Glasses 2 Refurbished Wireless”. A correction has been made to Section **5 Limitations**, paragraph 3 as below:

“Baseline pupil diameter values should be interpreted with caution, as calibration issues with the Tobii Pro Glasses 2 Refurbished Wireless and participant-specific factors may have influenced accuracy; future studies will address this with stricter pre-recruitment procedures, more controlled environments, and improved data collection protocols.”

The original version of this article has been updated.

