# Supplementary material for: Visual attention during non-immersive virtual reality balance training in older adults with mild to moderate cognitive impairment: an eye-tracking study
Source: Front Aging Neurosci. 2025 Oct 21;17:1671477. doi: 10.3389/fnagi.2025.1671477 (PMC12583179; doi:10.3389/fnagi.2025.1671477)
Supplement: Supplementary file 1 [file Data_Sheet_1.pdf]

Supplementary material

Table S1. Clinical metrics specification, tests, and description

Metrics	Test	Description	Cut-off/ Normal range
**Balance Level**	Mini-BESTest	It identifies whether a patient is at high or low risk of falling using 14 tests, each graded on a scale from 0 to 2 (0: fails the test; 2: passes the test). It comprises four dimensions: anticipatory postural control, reactive postural control, sensory orientation, and dynamic gait.	28 points, the cohort score for a low risk of falling will be around 19 points.
**Walking Stability**	Functional Gait assessment (FGA)	It indicates the level of gait stability through 10 tests over a distance of 8 feet.	30 points, the cohort score to categorize a stable walking will be about 19 points
**Walking Speed**	4-Meter Walk Test	In the geriatric population, this test determines whether the patient can walk independently within the home.	It is achieved if the patient reaches 0.8 m/s. A speed greater than 1.4 m/s is considered independent walking outside the home.
**Walking Endurance**	6-Minute Walk Test (6MWT)	Identify the number of meters walked in 6 minutes.	Normality is measured according to the patient's age, weight, and height.
**Cognitive level**	Montreal Cognitive Assessment (MoCA)	Cognitive screening of a patient in the following dimensions: visuospatial ability, executive function, attention, memory, temporal-spatial orientation, language ability, and abstraction.	30 points out of 26 points are considered a good cognitive level.

Non-Immersive Virtual Reality Exercise Performed			
Exercise	Description	Maximum Score	Difficulty
**Lateral Displacement**	Involves static marching where the patient alternately and randomly lifts the right or left foot	45 points.	Variations in lateralization randomness, stimulus frequency, and environmental

	when an obstacle appears.		opacity. The difficulty increases progressively based on patient performance.
**Walking Endurance**	Static Marching where the patient must avoid a randomly appearing player positioned in front, to the right or to the left.	60 points.	Adjust lateral randomness, stimulus frequency, and environmental opacity. The difficulty increases progressively based on patient performance.
**Single-Leg Support**	Patient must touch a ball on the ground with one foot, while the ball's position changes in distance.	21 points.	Variation in lateral randomness, stimulus frequency, and changes in the base support.

Metrics Considered for Non-Immersive VR Treatment	
**Training duration**	Measured in seconds
**Difficulty**	Assessed Based on changes in intensity, frequency, and time pressure.
**Performance**	Measured by the ratio of active time to accuracy percentage.
**Accuracy Percentage**	Number of correct repetition.
**Error Percentage**	Number of failed repetition.
**Response Time**	Measured in seconds.

Eye Tracker Metrics Considered	
Pupil Dimension Left, Right, Z	Refers to pupil size relative to the gyroscope data from the Tobii Glasses 2. An average pupil size is calculated for each participant from calibration through the recording session. Measured in millimeters (mm)
Pupil Diameter, Left, Right, Filtered	Refers to the pupil diameter as captured by the Tobii Glasses 2 at a given moment. Measurements in millimeters can be influenced by lighting conditions, cognitive load, or stimulus exposure.
Eye Movement Type	Identifies the classification of ocular movement detected at the millisecond level during recording.
	Saccade: A rapid eye movement lasting less than 20 milliseconds (200 microseconds), during which the pupil moves from point X to

	point Y.
Fixation	A relatively stable gaze where the pupil remains still or nearly still on a single point for more than 20 milliseconds.
Unclassified	Movements not categorized by the system, potentially due to lighting failure, participant pathology affecting the pupil, or abrupt head movements causing glasses misalignment.
EyesNotFound	Instances where the system could not detect the participant´s pupils, possibly due to sudden lighting changes or visual impairments affecting tracking.

Table S2. TREND Statement Checklist

Item No.	Descriptor	Reported in Page(s)	Included?
1	Title and Abstract	Page 1	✓
2	Background	Page 2	✓
3	Objectives	Page 2	✓
4	Participants	Pages 3–4	✓
5	Interventions	Page 4	✓
6	Outcomes	Pages 4–5	✓
7	Sample size	Page 4	✓
8	Assignment method	Page 3	✓
9	Blinding (masking)	Page 4 (Not applicable)	✗/✓
10	Unit of analysis	Page 4	✓
11	Statistical methods	Page 5	✓
12	Participant flow	Page 5, Table 2	✓
13	Recruitment	Page 4	✓
14	Baseline data	Page 5, Table 2	✓
15	Baseline equivalence	N/A in single-group design	N/A
16	Numbers analyzed	Page 5	✓
17	Outcomes and estimation	Page 5, Table 3	✓
18	Ancillary analyses	Not performed	✗
19	Adverse events	Page 6	✓
20	Interpretation	Pages 6–7	✓
21	Generalizability	Page 7	✓
22	Overall evidence	Pages 6–7	✓
